# Marine Collagen as A Promising Biomaterial for Biomedical Applications

**DOI:** 10.3390/md17080467

**Published:** 2019-08-10

**Authors:** Ye-Seon Lim, Ye-Jin Ok, Seon-Yeong Hwang, Jong-Young Kwak, Sik Yoon

**Affiliations:** 1 Department of Anatomy, School of Medicine, Pusan National University, Yangsan 50612, Korea; 2 Department of Pharmacology, School of Medicine, Ajou University, Suwon 16499, Korea

**Keywords:** marine collagen, scaffolds, 3D cell culture, tissue engineering, tissue regeneration

## Abstract

This review focuses on the expanding role of marine collagen (MC)-based scaffolds for biomedical applications. A scaffold—a three-dimensional (3D) structure fabricated from biomaterials—is a key supporting element for cell attachment, growth, and maintenance in 3D cell culture and tissue engineering. The mechanical and biological properties of the scaffolds influence cell morphology, behavior, and function. MC, collagen derived from marine organisms, offers advantages over mammalian collagen due to its biocompatibility, biodegradability, easy extractability, water solubility, safety, low immunogenicity, and low production costs. In recent years, the use of MC as an increasingly valuable scaffold biomaterial has drawn considerable attention from biomedical researchers. The characteristics, isolation, physical, and biochemical properties of MC are discussed as an understanding of MC in optimizing the subsequent modification and the chemistries behind important tissue engineering applications. The latest technologies behind scaffold processing are assessed and the biomedical applications of MC and MC-based scaffolds, including tissue engineering and regeneration, wound dressing, drug delivery, and therapeutic approach for diseases, especially those associated with metabolic disturbances such as obesity and diabetes, are discussed. Despite all the challenges, MC holds great promise as a biomaterial for developing medical products and therapeutics.

## 1. Introduction

Collagen, a biological macromolecule constituting 25% to 35% of protein in the human body, is the single most abundant mammalian protein and the key structural fibrous protein of the extracellular matrix (ECM) of biological tissues, in both invertebrate and vertebrate organisms [1,2]. Collagen is mostly present in fibrous connective tissues such as fasciae, aponeuroses, ligaments, tendons, periostea, perichondria, epimysia, perimysia, epineuria, perineuria, intervertebral discs, capsules of organs, adventitia of blood vessels and most hollow organs including gastrointestinal and genitourinary tracts, meninges, articular capsules, the dermis of the skin, and is also abundant in corneas, cartilages, bones, and sclerae, where it serves an essential structural role, providing tensile strength and flexibility to tissues and organs. At least 29 types of collagen have been identified so far and classified primarily according to their structure. Over 90% of the collagen in the body is type I, while the other common types of collagen include types II, III, and IV. Collagens are trimeric molecules composed of three polypeptide α-chains, have a characteristic triple helix-tertiary structure, and are rich in glycine, proline, and hydroxyproline residues.

The desirability of collagen and its wide applicability in various fields as a biomaterial principally depends on its immense properties such as biocompatibility, biodegradability, easy availability, and high versatility [3,4]. Furthermore, collagen can form a highly organized intricate three-dimensional (3D) architecture of woven fiber networks by self-aggregation and cross-linking. These networks resist tensile stress in multiple directions and support cell growth [5]. Thus, the importance of collagen is increasingly recognized as a key source of biomaterials in diverse areas ranging from injectable collagen solutions to biomimetic scaffolds for 3D cell culture, tissue engineering, drug delivery, and regenerative medicine [6,7]. In addition, its mechanical and biological properties also make it a nearly ideal choice as a biomaterial for cosmetic, pharmaceutical, and biotechnological applications [5,8]. Commercial collagen has been traditionally extracted mainly from terrestrial mammals, such as cattle and pigs, and is widely used in the food, cosmetic, pharmaceutical, and biomedical industries. However, outbreaks of bovine spongiform encephalopathy (BSE), transmissible spongiform encephalopathy (TSE), and foot-and-mouth disease (FMD) have increased the health concerns regarding the use of collagen and collagen-derived products from terrestrial animals in recent years [9]. In addition, its purification is difficult and expensive. Moreover, bovine collagen is prohibited in Hinduism and porcine collagen is prohibited in Islamic and Jewish cultures, due to religious beliefs [10]. Therefore, there is an urgent need to develop a source of collagen that is an alternative to that obtained from terrestrial mammals [11].

**Figure 1:**
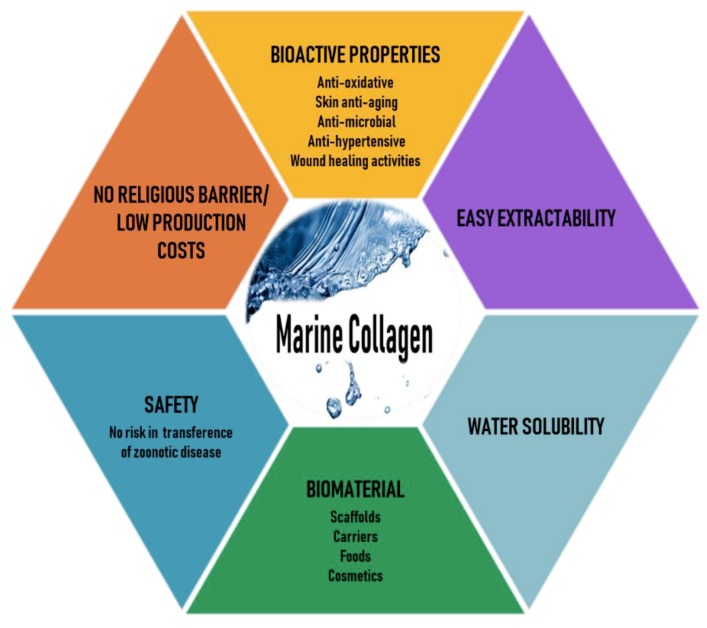
Various beneficial characteristics of marine collagen.

Marine organisms are a rich source of structurally novel and biologically active compounds. To date, many biological components have been isolated from various marine resources. Marine collagen (MC)—collagen derived from marine organisms such as fish, seaweeds, sponges, and jellyfish—offers advantages over mammalian collagen, as it can be easily extracted, is water-soluble, and is safe because it is free of the risks of animal diseases and pathogens such as those mentioned earlier, has better chemical and physical durability, and is available in abundant quantities [12,13]. Thus, recently, MC has attracted much attention as a mammalian collagen substitute, from biomedical researchers to the cosmetic, food, and nutraceutical industries [14,15,16]. The various beneficial characteristics of MC are illustrated in Figure 1.

Accordingly, this review focuses on the applications of MC in biomedical research with special reference to its role in the fabrication of biomimetic 3D scaffolds for 3D cell culture, tissue engineering, and regenerative medicine. The first section defines biomaterial scaffolds in biomedical applications. The second section describes the characteristics, isolation, and physical and biochemical properties of MC. Finally, the latest applications of MC-based scaffolds are discussed in detail.

## 2. Biomaterial Scaffolds in Biomedical Applications

A scaffold is a 3D structure manufactured from a variety of biomaterials and is a vital supporting element for cell attachment, growth, and maintenance in 3D cell culture and tissue engineering. The mechanical and biological properties of the scaffolds depend on the chemical composition, structure, and arrangement of their constituent macromolecules, and influence cell morphology, behavior, and function [17]. Scaffolds can be manufactured using a plethora of fabrication techniques. Ideally, scaffolds should not only provide a supporting structure, but also the chemical, mechanical, and biological cues required to respond to environmental stimuli. Unlike a 2D monolayer cell culture, a 3D cell culture is a much more satisfactory model that more accurately mimics in vivo characteristics of cell morphology, behavior, organization, and physiology, such as intricate cell–cell and cell–matrix interactions and complex transport dynamics for nutrients and cells [18]. 

When selecting a suitable scaffold for use in biomedical applications, a number of key factors should be considered, as follows: (1) The source of the biomaterial, (2) mechanical properties to support cells, (3) optimal biocompatibility to favor cellular attachment, viability, proliferation, growth, differentiation, and activity, and (4) fabrication methods that are available in a wide variety of shapes and sizes [19,20].

First, an appropriate biomaterial should be chosen for the fabrication of scaffolds. A biomaterial is any substance that has been engineered to interact with biological systems to support cells, enhance a biological function, or replace damaged tissue. Among the available types of biomaterials, polymers have been widely used for the fabrication of scaffolds due to their good processing characteristics. Typically, polymer scaffolds are in the form of hydrogels, fibrous meshes, or porous sponges. There are two types of polymers, synthetic and natural. Common synthetic polymers includes poly (ε-caprolactone (PCL), poly (lactic acid) (PLA), poly (glycolic acid) (PGA), and poly (lactic-co-glycolic acid) (PLGA), which are FDA approved, with good mechanical properties [21]. However, they have a disadvantage because, unlike natural polymers, they lack cell-binding sites due to their hydrophobic property. Alternatively, natural polymers, including polysaccharides (hyaluronic acid, alginate, chitosan, cellulose, and so on) and proteins (collagen, gelatin, fibrin, silk fibroin, keratin, and so on), are employed as favorable scaffolding materials. Unlike synthetic polymer-based scaffolds, natural polymers are usually biocompatible and biologically active and typically promote excellent cell adhesion and growth. However, natural polymers are often chemically modified to fabricate scaffolds because they generally do not have good mechanical properties and have a much lower thermal stability than synthetic polymers. Recently, researchers have shown an increasing interest in creating composite scaffolds made from a combination of two or more different phases of biomaterials, to improve their mechanical or biological properties and overcome the problems that exist in scaffolds fabricated from a single-phase biomaterial [22]. Thus, considerable research has been devoted to the development of composite scaffolds comprising of a number of phases for biomedical applications [23]. In particular, MC has recently emerged as a promising biomaterial with great potential in regenerative medicine and drug delivery applications because of its unique properties. This review outlines the properties of MC as a source of biomaterial and discusses approaches towards increasing the effectiveness of MC-based scaffolds in biomedical applications, either alone or with an additional phase incorporated to enhance biological and/or mechanical properties. 

Second, the scaffold should have good mechanical properties such as porosity, pore size, interconnectivity, stiffness, and tensile strength. The inter-connecting pores, with appropriate architecture and scale, within the scaffold are necessary to permit the proper diffusion of oxygen and nutrients, cell integrity and migration, and the sufficient stiffness, strength, and robustness of the scaffold to ensure that a scaffold does not collapse during handling, cell culture, and the host’s normal activities. These mechanical properties of scaffolds are critical for maintaining the structural stability of the biomaterial and for mimicking topological and microstructural characteristics of the extracellular matrix (ECM), thereby playing an important role in guiding cell growth and functions by regulating the interaction between cells and the diffusion of nutrients and metabolic wastes throughout the 3D constructs [24]. 

Third, a key functional requirement of any scaffold for 3D cell culture is that it must be biocompatible. Biocompatibility refers to the ability of a material to perform with an appropriate cellular or tissue response in a specific situation, without eliciting any undesirable local or systemic effects [25]. Biocompatible scaffolds must provide an optimal microenvironment for cell attachment, proliferation, migration, differentiation, and function. 

Finally, the most appropriate fabrication method should be selected based on the intended purpose [26]. The main approaches for manufacturing scaffolds for 3D cell culture can be broadly divided into the following two: The use of hydrogels and solid scaffolds. Hydrogels are used for the encapsulation of cells in a loose scaffold framework of a cross-linked natural base material with high water content. Hydrogels can be designed to support specific types of cell growth and function by either trapping cells in an artificial ECM protein environment or by allowing cells to migrate into the interior of the gel from the surface [27]. A unique feature of hydrogels is that cells grow as isolated aggregates within the gel. Cells may be encapsulated into the gels by self-assembly, physical cross-linking, ionic cross-linking, or radical polymerizations by UV exposure, although the use of a chemical cross-linker and UV light to cure the gel may exert a detrimental effect on cells [28]. 

Solid scaffold-based technology also provides a 3D space to support cells, allowing them to create natural 3D tissue-like structures. Solid scaffolds are broadly divided into fibrous or porous matrices manufactured using a range of different techniques and materials. One of the primary reasons for using solid scaffold-based technologies is their ability to produce organized arrangements of cells in vitro, in a controllable manner. For example, electrospinning produces a mesh of fibers that allow cells to adhere and elongate along the fibers, which induces cell alignment and directionality to the cultures [29,30]. Solid porous scaffolds have been used for the co-culture of different cell types in layered arrangements in close proximity [27].

The two main scaffolds used for biomedical applications so far are biomaterial scaffolds and biological scaffolds, with the former typically composed of synthetic or natural polymers and the latter usually derived from the ECM of intact mammalian tissues through decellularization. Both types of scaffolds have a 3D topological architecture that can closely mimic the native ECM. However, biomaterial scaffolds commonly offer greater value compared to biological scaffolds with regard to critical properties of scaffolds, such as architecture, pattern, biocompatibility, porosity, stiffness, and modulation of degradation rate [31,32,33]. Biomaterial scaffolds provide specific microenvironmental cues in a 3D controlled fashion, not only to support embedded cells and enhance cell survival, infiltration, and differentiation, but also to facilitate cell-ECM interactions, thereby rendering them useful as an artificial ECM in a variety of biomedical applications including in vitro 3D cell culture, tissue engineering, and regenerative medicine. They have also been applied for the controlled release and delivery of drugs and therapeutic biomolecules, cell transplantation, and 3D bioprinting [33,34].

**Figure 2:**
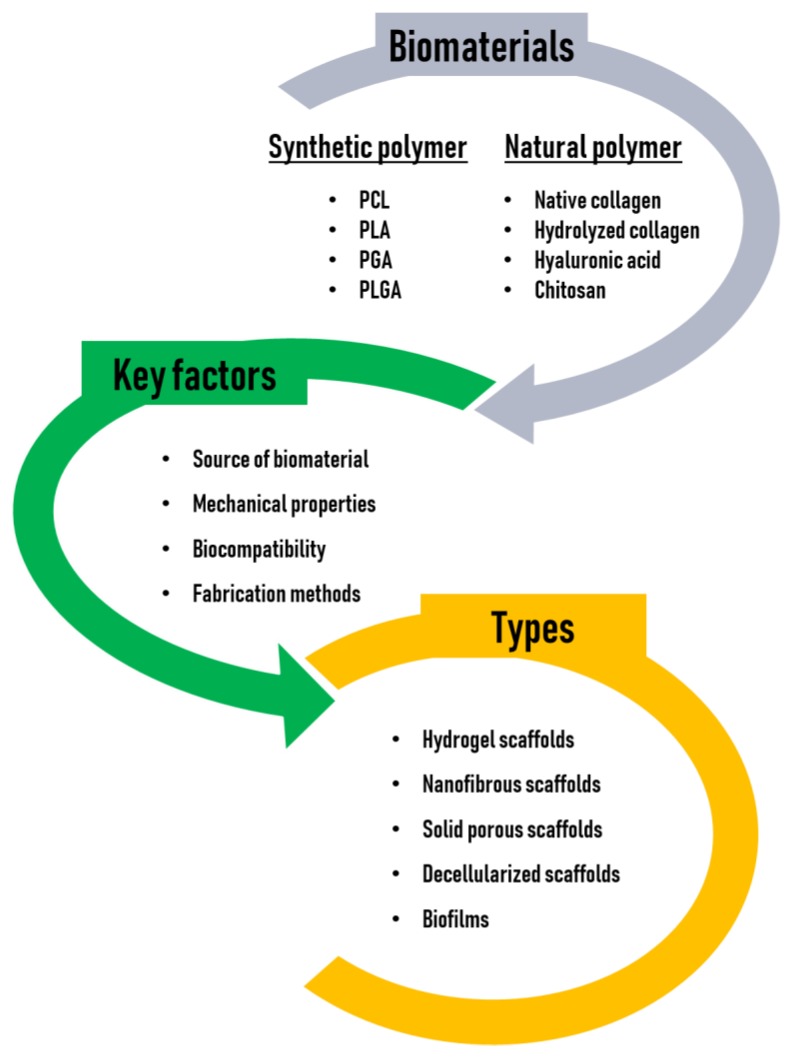
Biomaterials, key factors, and types of scaffolds for tissue engineering.

Tissue engineering scaffolds may be conductive or inductive. Conductive scaffolds, which have been widely used, provide and maintain a 3D environment that supports passive cell infiltration, thereby creating a pseudomicroenvironment, although this passive structural element does not provide sufficient information to promote full tissue regeneration in most applications. On the other hand, inductive scaffolds are designed to more closely mimic the native cellular microenvironments and may contain a wide variety of naturally occurring bioactive molecules or synthetic analogs of structural, functional, or specialized proteins and proteoglycans in the body [35]. In this regard, the tissue engineering field is transitioning to inductive scaffolding, but this approach is still faced with many problems such as time-consuming processes, shortage of cell sources, uncertain regulation of cell differentiation, the risk of host immune reactions, difficult storage and transportation of the constructs, and difficulty in controlling the generation or regeneration of functional native tissues. Thus, it presents significant challenges [36]. The biomaterials, key factors, and types of scaffolds for tissue engineering are illustrated in Figure 2.

## 3. Collagen Derived from Marine Organisms

### 3.1. Characteristics of MC

The ocean covers over 70% of the earth’s surface and marine species comprise approximately half the total global biodiversity, so it serves as an enormous resource that provides collagen and other natural substances that can be utilized for the development of various cosmetic, nutraceutical, food, pharmaceutical, medical, and biomaterial products. This diversity in the marine environment signifies the development and application of novel MC-based chemobiological molecules and bioactive proteins and peptides for biomedical applications [37,38,39,40]. 

MC can be isolated from marine invertebrates (sponges, jellyfish, sea urchin, octopus, squid, cuttlefish, sea anemone, prawn, star fish, and so on), marine vertebrates (fish and marine mammals) [41,42,43], and other marine sources such as algae. MC has attracted wide scientific and industrial interest due to its water solubility, safety, biocompatibility, high biodegradability, low immunogenicity, easy extractability resulting in high yield, and low production costs [44]. Due to the enormous amount of fish wastes being discarded in the form of skins, bones, fins, heads, guts, and scales, finding adequate modalities for converting these marine waste residues into useful products of high significance and economic value is of essence [45]. Collagen extracted from marine sources, typically used as important biomaterials for the fabrication of scaffolds, is predominantly type I collagen, whereas type II and type IV collagens can be isolated from fish cartilage and some jellyfish and marine sponges, respectively [41,46].

### 3.2. Isolation of MC

The preparation of MC involves cleaning, separation, and size reduction of the samples, followed by a chemical pre-treatment to remove non-collagenous proteins, pigments, or fats. Since the composition and structure of the ECM is different across different regions of the marine animal, the applied MC extraction methodology must include desirable preparation processes. Since MC is poorly soluble in cold water due to the presence of strong cross-links in its triple helix structure, a mild chemical treatment using either diluted acids or bases is a prerequisite to break the cross-links for MC extraction. Acidic pretreatment cleaves non-covalent inter-and intra-molecular bonds, while alkaline pretreatment removes non-collagenous proteins and pigments without causing structural modification of the collagen chains [47]. The common method to remove non-collagenous proteins is the use of sodium hydroxide (NaOH). The removal of fats and pigments can be achieved by using alcohols, such as butyl-alcohol or ethanol, and oxygen peroxide, respectively [14,48]. For MC extraction from skeletal tissues such as bone, cartilage, and scales, ethylenediaminetetraacetic acid (EDTA) or hydrochloric acid (HCl) is required for demineralization purposes [49]. 

The most commonly used dilute acid for MC extraction is acetic acid, while other acids such as HCl, citric acid, and lactic acid are also employed. Higher extraction yields of collagen from codfish skin were obtained using acetic and lactic acids, while the typical acid solubilization method resulted in a low collagen yield [50]. To overcome this obstacle, proteolytic enzymes such as pepsin, trypsin, papain, alkaline protease, bromelain, pancreatin, or alcalase, have been utilized because of their ability to help in the solubilization process by specifically cleaving peptides in the telopeptide region of collagen, which are the non-helical ends [51]. Among these enzymes, pepsin is the most commonly used enzyme for MC extraction. In addition, enzyme treatment can reduce the antigenicity caused by telopeptides [52]. Besides telopeptides, antigenicity related to noncollagenous proteins, cells, and cell remnants can be removed by the above-mentioned method of NaOH treatment [53]. Finally, the solubilized collagen is centrifuged and salted out by adding sodium chloride (NaCl) and then the precipitate is dialyzed and freeze-dried.

Collagen extracted using acid solution is referred to as acid-soluble collagen (ASC) and collagen extracted using pepsin is called pepsin-soluble collagen (PSC) or atelocollagen. Generally, it is common to use this proteolytic procedure after the extraction of ASC [51]. Marine invertebrates that contain too much water in their body, such as jellyfish, exhibit poor collagen solubility in acetic acid. In such cases, homogenization or freeze-drying is needed to improve the efficiency of collagen extractability [47,54]. Collagen from echinoderms such as sea urchins cannot be extracted by the traditional acid solubilization method. The extraction of intact collagen fibrils from sea urchin was developed by the group of Sugni et al. using a hypotonic solution and a SDS-based decellularizing solution [55,56]

**Figure 3:**
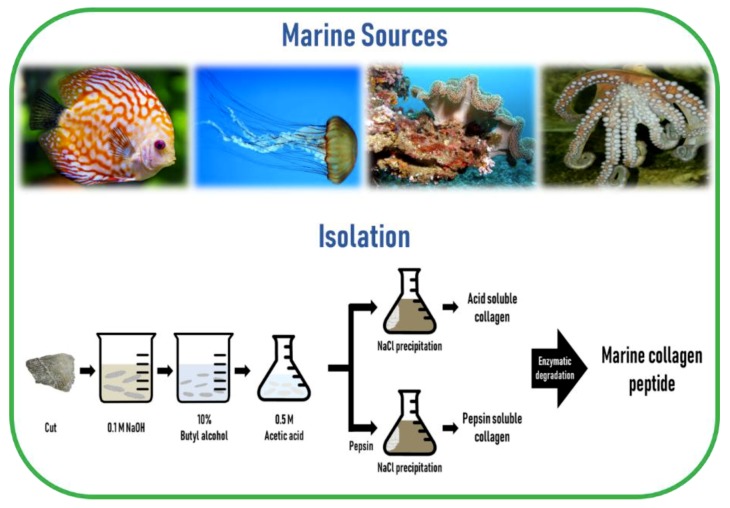
A schematic diagram of marine collagen isolation from marine sources.

Hydrolyzed collagen (also called collagen hydrolysates or collagen peptides) is a polypeptide composite made by further acidic or enzymatic hydrolysis of collagens, but enzymatic hydrolysis is generally preferred, as it is safer, cheaper, more moderate, and less destructive than acid hydrolysis [57,58]. The molecular weight of hydrolyzed collagen is generally in the range of approximately 500–25,000 Da [59]. Hydrolyzation does not damage the integrity of the triple helix of collagen, thereby preserving the tropocollagen akin to the collagen in the living tissue. Furthermore, marine collagen peptides (MCP) are safer than those obtained from mammals, are biologically active, have improved chemical and physical properties, and an enhanced function [60,61], as well as a number of bioactive properties such as anti-oxidative, skin anti-aging, anti-microbial, anti-hypertensive, and wound healing activities, compared to their non-hydrolyzed form [51]. The schematic diagram of marine collagen isolation from marine sources is illustrated in Figure 3.

It is critical to limit the enzyme activity to prevent excessive degradation of collagen. Enzyme inactivation can be achieved by either altering the pH of the extract solution, or by dialyzing the extract solution against buffer solutions [42]. Since different extraction methods for MC may affect the quality of products, efforts have been made which aim to isolate MC with high purity, high yield, reserved structural integrity, and its unique properties including gel-forming capacity, water-retaining capacity, and thermal stability [62].

### 3.3. Physical and Biochemical Properties of MC

Type I MC is unique in terms of its extremely high solubility in dilute acid compared to collagen from terrestrial animals [63,64]. Compared with terrestrial animal-derived type I collagen, type I MC derived from bony fish has been found to exhibit a high degree of structural similarity between species with respect to the α1 and α2 chains [12].

The biochemical composition of MC is thought to be different from that of mammalian collagen. For biochemical analyses, the application of strict conditions for sample preservation prior to collagen extraction is indispensable, as the stability of the hydroxyproline content strongly depends on the sampling procedure. Several previous studies have demonstrated that the amino acid composition of MC is similar to that of mammalian collagen [65,66,67,68,69]. Glycine is the most abundant amino acid, accounting for more than 30% of all amino acids [12]. Generally, the content of imino acids, including proline and hydroxyproline, is lower in MC than that of mammalian collagen [70], but contains more serine and threonine residues, particularly in fish collagen derived from cold water species [71]. These differences in amino acid composition, especially in terms of hydroxyproline content, are generally responsible for differences in collagen properties, such as rigidity, temperature stability, and denaturation temperature [72,73]. A high imino acid content is needed for the stabilization of collagen [74]. 

On the other hand, collagen derived from warm water fish species, including tilapia, can exhibit similar amino acid composition, rheological properties, and thermostability to that of mammalian collagen [75], suggesting that tilapia collagen can be used as an alternative to mammalian collagen in biomedical applications. The hydroxyl groups of hydroxyproline and hydroxylysine increase the thermal stability of the collagen triple helix by interchain hydrogen bonding via a bridging water molecule and by direct hydrogen bonding to a carbonyl group [76]. Therefore, the extent of hydroxylation of proline and lysine directly influences the thermal stability of collagen and the higher the content of imino acids, the higher the thermal stability of collagen [77]. Generally, the denaturation temperature (Td) of MC is lower than that of mammalian collagens, due to its lower content of imino acids [78]. Therefore, differences in hydroxyproline content might determine the Td of collagens from different species. Collagens derived from fish living in cold environments have lower contents of hydroxyproline and they exhibit lower thermal stability than those from fish living in warm environments [77]. It was found that the degree of hydroxylation of proline in cold sea fish, for example, chum salmon, has been reported to be relatively low (35–37%) compared to that of warm sea fish (e.g., tilapia: 43%). Therefore, the imino acid content of fish collagens is associated with their thermal stability and correlates with the water temperature of their normal habitat [79]. Since the Td of MC is lower than the mammalian body temperature, MC melts when placed in temperatures greater than 37 °C. 

Manufacturing of scaffolds synthesized from MC, especially collagen from fish, is hindered by the lower Td and viscosity than land vertebrate collagens [78]. Furthermore, improvements in collagen fibrillogenesis can be achieved with chemical cross-linking in vitro. Numerous cross-linking methods have been employed to stabilize collagen. The cross-linking methods employed to stabilize collagen can be divided into physical treatments, such as UV irradiation, gamma irradiation, and dehydrothermal treatment and chemical treatments, such as those involving the use of glutaraldehyde, carbodiimide, or 1-ethyl-3-(3-dimethyl-aminopropyl)-carbodiimide (EDC). Chemical treatments confer remarkably high strength and stability to the collagen matrix, although they can result in potential cytotoxicity or poor biocompatibility, whereas physical treatments provide sufficient stability with no cytotoxicity.

## 4. Biomedical Applications

**Figure 4:**
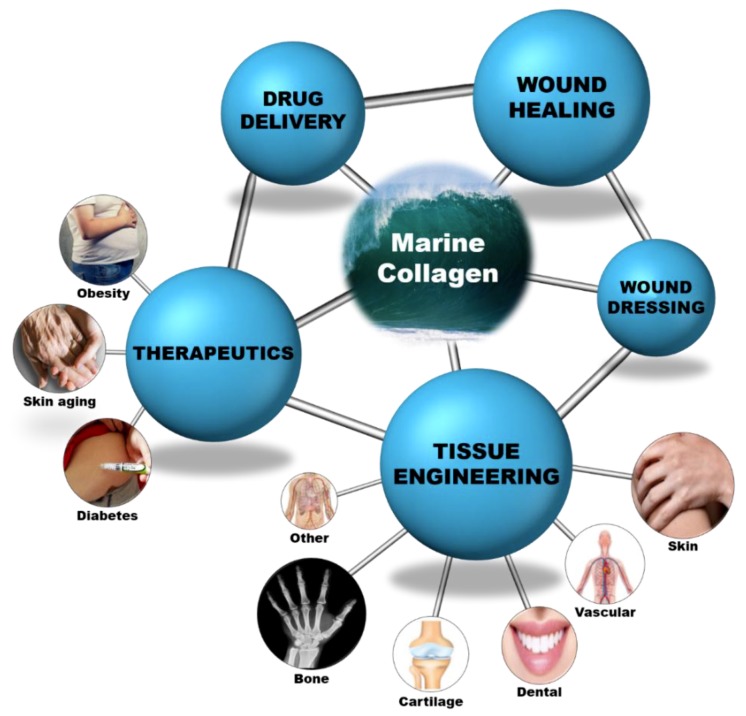
Marine collagen as a biomaterial for biomedical applications.

Collagen is a versatile biomaterial that finds application in various fields and its wide applicability in the medical field is revealed in detail in this review. Since it exists in various forms, it plays a vital role in the biomedical field. The major biomedical field applications are shown as a schematic layout in Figure 4 and their detailed descriptions are summarized in Table A1, Table A2, Table A3, Table A4, Table A5, Table A6, Table A7, Table A8, Table A9 and Table A10. Various formulations and their respective applications are detailed as follows:

### 4.1. Tissue Engineering and Regeneration 

Tissue engineering or regenerative medicine is an emerging and rapidly developing interdisciplinary field of life science, employing both engineering and biological principles to create new tissues and organs, to promote the regeneration of damaged or diseased tissues and organs by combining cells from the body with highly porous scaffold biomaterials. The excellent biocompatibility of MC has provoked its potential role in tissue engineering and in regenerative medicine for the design of biomaterial scaffolds. 

#### 4.1.1. Bone Tissue Engineering and Regeneration 

Repair of bone fractures and fracture healing are postnatal regenerative processes that recapitulate many features of bone development and can be considered as forms of tissue regeneration. Hence, bone regeneration is a complex physiological process of bone remodeling, which involves bone formation and bone resorption. However, there are complex clinical conditions in which bone regeneration is required in large amounts, such as for skeletal reconstruction of large bones due to defects and damage by trauma, infection, tumor resection, or skeletal abnormalities, or cases in which the regenerative process is compromised, including avascular necrosis, atrophic non-unions, and osteoporosis [80]. Currently, the advances in tissue engineering technology have begun to revolutionize the methods to repair or regenerate bones, create bone grafts, or substitutes utilized in a wide array of relevant clinical settings [81]. At present, there is an exceedingly high demand for functional bone grafts worldwide. In the United States, annually, more than half a million patients receive bone repairs, with a cost greater than $2.5 billion [82].

During the past decade, MCP have been examined to have a substantial osteogenic activity [83]. MCP extracted from the scales of tilapia induced multidirectional differentiation in the primary rat bone marrow mesenchymal stem cells, where MCP not only promoted cell viability, but also significantly upregulated the expression of osteogenic markers, as well as endothelial markers, suggesting that MCP has the potential to actively promote osteogenic and endothelial differentiation. Interestingly, it was also shown that MCP inhibited the expression of adipogenic and chondrogenic markers [84]. Yamada et al. [85] designed a study to investigate the biological effects of MCP extracted from the bone and skin of cods on human osteoblastic cells (NOS-1). It was found that treatment with MCP increased cell proliferation, expressions of osteogenic markers, alkaline phosphatase activity, and mineralization, indicating the potential utility of MCP as a biomaterial in osteoblast cell cultures for bone tissue engineering. The effects of MCP purified from Gadiformes and Pleuronectidae on collagen synthesis, quality, and mineralization in a mouse osteoblastic cells (MC3T3-E1) culture system were observed [86]. Results from this study showed that collagen treatment accelerated matrix mineralization and collagen deposition in cultures and significantly upregulated the gene expression of several collagen modifying enzymes, thereby suggesting the potential utility of MCP in bone tissue engineering. The effect of MCP on osteoblastic differentiation of human mesenchymal stem cells (hMSCs) was increased adherence, which remarkably accelerated the early stage of osteoblastic differentiation with upregulated osteoblastic markers, suggesting that MC is a facilitator of osteoblastic differentiation [87].

The effect of MCP derived from chum salmon (*Oncorhynchus keta*) skin on the development of femurs in growing rats of both sexes has been investigated [88]. The data from this study supported that MCP supplementation increased not only serum osteocalcin and bone-specific alkaline phosphatase content, but also the size, mineral density, dry weight, ash weight, content of most minerals, and both stiffness and toughness of femurs in growing male rats, suggesting that MCP could promote the development of long bones in growing male rats. MCP extracted from scales of two kinds of fish, Sparidae and Chanos, promoted the proliferation of osteoblasts and inhibited the proliferation of mature osteoclasts [89]. These findings indicate MCP possesses a high biological activity to promote bone tissue regeneration by stimulating the osteogenic potential of osteoblasts and prevent osteoporosis by regulating osteoclast activity.

Numerous studies have revealed that MC-based biomaterial scaffolds have been used as bone tissue substitutes and reinforcements that promote bone tissue regeneration. The development of scaffolds to promote cellular growth inside them has been one of the fundamental goals of bone tissue engineering [24,90]. A nano/micro fibrous scaffold, through self-assembly by utilizing collagen extracted from scales of fresh water fish, *Labeo rohita* (Rohu) and *Catla catla* (Catla), was fabricated via a freeze-drying method for the purpose of application in tissue engineering [91]. In this study, minimal inflammatory response was elicited when the collagen solution was injected in mice and the nano/micro fibrous scaffold exhibited considerable cell viability and induced significant proliferation rate of human osteoblast-like cells (MG-63) and mouse fibroblasts (NIH3T3), suggesting that the MC-based scaffolds are biocompatible in nature and may have potential tissue engineering applications.

In line with this finding, a wide variety of collagen-based composite scaffolds mimicking the native bone tissue microenvironment have been proposed by applying diverse materials to modify collagen-based scaffolds for better performances in vitro and in vivo, thereby accelerating the bone tissue regeneration. A MC/apatite composite scaffold was fabricated via an optimized method of freeze-drying using collagen from shark skin (*Prionace glauca*) and apatite from shark teeth of *Isurus oxyrinchus* and *Prionace glaucabiphasic* for hard tissue applications [92]. This MC/apatite composite scaffold efficiently promoted the viability of human osteosarcoma cells (Saos-2), suggesting its role as a potential structure for bone regeneration therapeutic approaches. For a different approach in the context of designing MC/apatite-based composite scaffolds, Pallela et al. [93] incorporated irciniid collagens (from marine sponge, *I. fusca*) along with natural hydroxyapatite (from *Thunnus obesus* bone) and chitosan matrix, for chitosan-hydroxyapatite-marine sponge collagen scaffold fabrication as a potential matrix for bone regeneration. As a result, this MC/hydroxyapatite-based scaffold exhibited enhanced porosity and thermal stability and an increased cell proliferation of human osteoblast-like cells (MG-63), suggesting its potential prospects in the field of bone tissue engineering. In the same way, a collagen/hydroxyapatite/chitosan composite sponge was developed via lyophilization using type II collagen from blue shark (*Prionace glauca*) cartilage for guided bone tissue engineering. It was demonstrated that this MC/hydroxyapatite-based composite sponge mineralized with hydroxyapatite efficiently promoted the viability of osteoblast cells (hFOB12) and significantly elevated osteoblastic alkaline phosphatase activity, suggesting its role as a suitable biomaterial for a bone tissue engineering application as an alternative to mammalian collagen scaffolds [94]. 

For another attempt, a MC/glycosaminoglycan (GAG)/Aquamin composite scaffold was manufactured via lyophilization of MC/GAG suspension from shark cartilage for the development of a MC-based scaffold that could be used as a bone graft substitute with improved mechanical properties [95]. Mouse pre-osteoblastic cells (MC3T3-E1) cultured on the MC/GAG/Aquamin composite scaffolds showed improved osteogenesis as measured by alkaline phosphatase, osteopontin, and osteocalcin expression, which was further confirmed by increased mineralization as determined by von Kossa and Alizarin red staining, indicating a significant potential for facilitating bone repair in vivo. In addition, a composite disk was synthesized via a vacuum-drying method using marine sponge collagen, hydroxyapatite, and poly (methyl methacrylate) (PMMA) for bone regeneration applications [96]. In this study, marine sponge collagen was shown to promote cell viability of mouse pre-osteoblastic cells (MC3T3-E1) and murine fibroblasts (L929), suggesting that marine sponge collagen can be used as the organic part of an artificial bone graft with improved biological properties. 

Mredha et al. [97] recently developed a novel class of collagen fibril-based tough double network composite hydrogels using collagen extracted from Bester sturgeon fish and poly (N,N′-dimethylacrylamide) (PDMA). This scaffold exhibited excellent biomechanical performance in vivo after 4 weeks implantation of the gels in the osteochondral defect of rabbit knee, suggesting its potential use in designing next-generation orthopedic implants such as artificial cartilage and bone defect repair material in the load-bearing region of the body. Fabrication of a biphasic scaffold from mineralized salmon collagen, alginate, and fibrillized jellyfish collagen was conducted via joint freeze-drying and chemical cross-linking method, aiming at the repair and regeneration of osteochondral defects [98]. This biphasic MC-based scaffold supported the chondrogenic and osteogenic differentiation of bone marrow-derived hMSCs, indicating its potential as a suitable scaffold for the development of osteochondral constructs. Furthermore, a MC/nanohydroxyapatite/PLGA composite nanofibrous membrane was prepared by electrospinning using fish scale and skin collagen and nanohydroxyapatite enhanced PLGA for guided bone regeneration [99]. The presence of MC in this membrane significantly enhanced the mechanical strength and accelerated the degradation rate and augmented cytocompatibility with bone mesenchymal stem cells and human gingival fibroblasts, indicating a remarkable potential for guided bone or tissue regeneration. 

Taken together, these data support the view that MC-based biomaterial scaffolds are playing an increasingly important role in bone tissue engineering and regeneration.

#### 4.1.2. Cartilage Tissue Engineering and Regeneration 

Articular cartilage damage is among the most encountered musculoskeletal diseases, eventually leading to total joint replacement if not treated properly. The cartilage has a very limited ability to self-regenerate, due to its avascular structure. Thus, the field of articular cartilage tissue engineering, which aims to repair, regenerate, and/or improve injured or diseased articular cartilage functionality, has garnered intense interest and holds great potential for improving articular cartilage therapy. 

It has been found that MCP from the skin of deep-water ocean fish (cod, haddock, and pollock) has the potential to enhance chondrogenic differentiation of primary adipose-derived stromal cells [100]. Oral administration of MCP from skins of Gadiformes species and glucosamine effectively controlled cartilage degradation in osteoarthritis-induced rabbit models, suggesting that MCP can be of potential interest as a disease-modifying agent for the prevention of degenerative joint disease [101]. In line with these findings, MCP was shown to promote chondrogenesis of hMSCs, resembling the effect of MCP on osteoblastic and osteogenic differentiation of hMSCs [102]. Hsu et al. [102] showed that hMSCs cultured with MCP showed increased expression of chondrogenic markers and decreased expression of osteogenic markers, indicating that MCP may provide the appropriate signals for the chondrogenic differentiation of hMSCs in vitro. These data support the application of fish collagen in cartilage repair. MC-based scaffolds synthesized via the freeze-drying method using collagen harvested from the jellyfish (*Rhopilema esculentum*) were shown to be favorable for the proper growth of primary human and rat nasal septum chondrocytes in a 3D environment where chondrocytes adhered to MC-based scaffolds and produced cartilaginous matrix proteins [103]. In this study, MC-based scaffolds were able to prevent septal perforations in an orthotopic rat model, suggesting that this scaffold is suitable for nasal cartilage repair. The potential therapeutic application in articular cartilage repair was uncovered recently using hydrogel hybrid scaffolds constructed from fibrillized jellyfish (*Rhopilema esculentum*) collagen and alginate [104]. The authors revealed that the hybrid scaffold increased the expression of chondrogenic hMSCs, suggesting that the hybrid construct supports hMSC chondrogenic differentiation and provides a novel direction in regenerative cartilage research. Clinical studies proved that MCP promoted cartilage matrix synthesis and reduced pain in osteoarthritic patients, making them a promising candidate for therapeutic agents against human osteoarthritis and osteoporosis [105].

#### 4.1.3. Skin Tissue Engineering, Regeneration, and Wound Healing

Despite its clinical importance, skin grafts have many limitations, including the availability of the donor. Over the centuries, an increasingly strong demand of skin substitutes for the replacement of skin defects resulting from burns, trauma, infection, graft rejection, scarring, genetic defects, and other diseases became a major healthcare challenge. The normal wound healing process in humans can be divided into three phases, as follows: Inflammatory, proliferation, and maturation. During inflammation, permeability is increased to enhance adhesion of cells to allow for the contraction of wound edges during proliferation and finally, during maturation, regression and differentiation take place to allow for the formation of new capillaries and fibroblast differentiation [106]. Furthermore, for wound healing to proceed, foreign particles and nonviable tissue should be removed from the wound surface [107]. Effective wound healing is essential to enable maintenance of homeostasis and to prevent penetration of infectious agents [108,109]. 

The wound healing effect of poly (3-hydroxybutyrate-co-4-hydroxybutyrate) (P(3HB-co-4HB)) scaffolds where the aminolyzed surface was conjugated by MCP from tilapia skin was described by Vigneswari et al. [110]. The P(3HB-co-4HB)/MCP scaffolds enhanced the attachment and proliferation of mouse fibroblasts (L929) and had a significant effect on wound contraction and wound closure, suggesting that this scaffold may serve as a future platform for wound healing technologies. Collagen from mrigal fish (*Cirrhinus cirrhosis*) scales was used for the fabrication of collagen scaffold via the freeze-drying method [111]. This MC sponge supported efficient growth and proliferation of primary human fibroblasts and keratinocytes and 3D co-culture with fibroblast and keratinocyte cells on this scaffold resulted in faster wound healing and the development of a stratified epidermal layer in vitro, proving its applicability as a dermal substitute. Ullah et al. [112] fabricated a composite fish collagen/chitosan/glycerin porous scaffold using collagen from tilapia fish scales via the freeze-drying and dehydrothermal (DHT) cross-linking method and found that the scaffold promoted human fibroblasts and keratinocytes proliferation, attachment, and infiltration in vitro, suggesting the scaffold can be effectively used for skin tissue engineering and regeneration. An orientated collagen matrix gel was fabricated by a simple centrifugation method and EDC cross-linking using PSC isolated from the skin of *Leiocassis longirostris* [113]. This structurally oriented collagen matrix facilitated the proliferation and migration of mouse fibroblasts (NIH3T3), suggesting future possibilities for different applications in skin wound healing and tissue engineering. A fish skin collagen/alginate/chitooligosaccharide integrated scaffold was fabricated via the blending and freeze-drying technique [114]. The human dermal cells (NHDF-neo) cultured on the composite scaffold exhibited excellent cell adhesion and proliferation, indicating the suitability of the scaffold as a superior candidate for skin tissue engineering applications. A biocompatible scaffold produced from pepsin-solubilized tilapia (*Oreochromis niloticas*) fish scale collagen, in the form of a hydrogel, facilitated active proliferation of baby hamster kidney cells (BHK-21), suggesting that tilapia scales can be an effective source of collagen extraction for use as a potential biomaterial in skin regeneration and tissue engineering applications [115].

When the in vivo wound healing properties of tilapia skin collagen and bovine collagen were compared, MC showed similar stimulating effects as bovine collagen in wound contraction, fibroblasts proliferation, collagen synthesis, re-epithelialization, and dermal reconstitution, along with the upregulated expressions of epidermal growth factor, fibroblast growth factor (FGF), and vascular endothelial marker [116]. Moreover, the authors demonstrated that PSC and ASC sponges synthesized from tilapia skin also have similar wound healing abilities. These results indicate that PSC and ASC as well as bovine collagen could be interchangeably used as biomaterials for developing bioactive surgical compresses or dermal substitutes to accelerate wound healing or treat severe wounds. A collagen/chitosan sponge was fabricated using collagen from the skin of a weever via lyophilization and EDC cross-linking with pressing for use as a dural substitute [117]. This scaffold not only provided excellent biocompatibility for the activity and proliferation of the mouse embryonic fibroblasts (MEFs) in vitro, but also prevented brain tissue adhesion, reduced inflammation, facilitated growth of fibroblasts, and enhanced the tissue regeneration and healing in the in vivo rabbit dural defect model, suggesting that the MC-based scaffolds can be used efficiently as engineered dural substitutes. Hu et al. [118] isolated MCP from the skin of Nile tilapia (*Oreochromis niloticus*) and evaluated its wound healing activity in vitro and in vivo and demonstrated that MCP enhances the scratch closure of human keratinocytes (HaCaT) in vitro and facilitates the process of wound healing in the deep partial-thickness scald wound rabbit model, supporting its potentially promising applications in wound care. 

In a rat model of uterine wound scarring and repair, MCP from chum salmon skin enhanced skin tensile strength and uterine bursting pressure, increased hydroxyproline levels, and facilitated the formation of capillaries, fibroblasts, and collagen fibers [119]. This study reported an improved synthesis of collagen and ECM components via an enhanced expression of transforming growth factor-β1 (TGF-β1) and basic fibroblast growth factor (bFGF), respectively, and alleviation of inflammation by upregulation of the expression of cluster differentiation 31 (CD31), following the administration of MCP, demonstrating its wound healing potential. Zhang et al. [120] also reported that oral administration of MCP from chum salmon skin enhanced cutaneous wound healing and angiogenesis in rats through the upregulation of vascular endothelial growth factor (VEGF) and FGF-2, demonstrating the potential role of MCP as a therapeutically beneficial biomaterial to treat wounds in clinical practice.

#### 4.1.4. Wound Dressing and Skin Repair 

The skin functions as a protective barrier and a breakdown in this function results in a wound injury. The treatment of a wound injury is to rapidly restore the construction and function of the wound to the levels of normal tissue. Traditional medicine for the treatment of skin wounds dates back to ancient times, however, traditional medicine dressing is not able to rapidly restore the wound [121]. Therefore, the search for new medical tissue engineering materials to function as a temporary barrier in damaged skin while avoiding the wound being infected is a foremost aim. In this regard, considerable attention has been given to the utilization of MC as wound care dressings.

A multifunctional and biomimetic tilapia skin collagen/bioactive glass nanofiber was fabricated via electrospinning for use as a skin wound dressing to protect against infection, to promote wound healing, and to enhance skin regrowth [122]. This composite nanofiber showed excellent anti-bacterial activity against *Staphylococcus aureus*, promoted the adhesion, proliferation, and migration of human keratinocytes (HaCaT), induced the secretion of type I collagen and VEGF by human dermal fibroblasts, stimulated the proliferation of human vascular endothelial cells, and accelerated skin wound healing in a rat wound model, providing the possibility of using it as a functional skin wound dressing. New bio-based topical formulations, in the form of powder or polymeric film, were developed using a natural horny skeleton of marine sponges (Porifera, Dictyoceratida). The marine sponge collagenic skeleton-based film was able to absorb the excess wound exudate, suggesting the beneficial role of natural sponge skeletal scaffold as an effective regulator of wound healing processes [43]. A carboxymethyl guar gum scaffold film grafted with ethylenediamine and MCP, cross-linked with ceftazidime, was synthesized by Jana et al. [123] for wound dressing applications. This aminated film exhibited excellent biocompatibility with mouse fibroblasts (NIH3T3) and displayed anti-microbial activity against *Staphylococcus aureus* and *Pseudomonas aeruginosa*, demonstrating its efficiency as a bioactive carrier for the regulation of wound healing processes. Plant extracts possessing active ingredients have been utilized to construct MC-based scaffolds for better performance against bacterial infections. A porous collagen sponge was prepared from fish (*Lates calcarifer*) scales incorporated with extracts of *Macrotyloma uniflorum* to impart anti-microbial activity to the sponges [124]. This collagen sponge showed excellent biocompatibility in the culture of mouse fibroblasts (NIH3T3) and human keratinocytes (HaCaT), suggesting that the sponge may be a potential candidate for the burn/wound dressing material. A composite electrospun nanomembrane of MCP/polyvinyl alcohol (PVA)/chito-oligosaccharides not only showed good anti-bacterial activities against *Staphylococcus aureus* and *Escherichia coli*, but also showed good biocompatibility in vitro and supported the proliferation of human skin fibroblasts, indicating its potential application in wound dressings [125].

Moreover, MC has been increasingly utilized for the development of cosmeceutical products due to its abilities in skin repair. Clinical trials showed that ingestion of MCP for 6 weeks improved skin hydration in woman volunteers, demonstrating the beneficial effects of MCP on ageing skin [126,127]. Kalil et al. [128] aimed to evaluate skin changes associated with the use of orthosilicic acid stabilized by MCP. Clinical evaluation results demonstrated no side effects, hypersensitivity, or systemic effects, and exhibited not only excellent changes in skin texture, firmness, and hydration, but also enhanced brightness and overall appearances in the MCP treated group. This suggests the potential role of MCP in therapeutic skin rejuvenation applications. Currently, MCP isolated from the marine sponge were shown to stimulate cell survival and proliferation of mouse macrophages (RAW 264.7), mouse fibroblasts (L929), and human keratinocytes (HaCaT), demonstrating significant anti-oxidant activity and protecting cells from UV-induced apoptosis, indicating that MCP have wound healing and photoprotective properties [129]. In addition, this finding suggests the potential application of MCP in drug and cosmetic formulations for damaged or photoaged skin repair. 

Therefore, due to their dual properties in wound healing and anti-microbial activity, MC-based scaffolds could be used as optimal dressings suitable for wound management and care.

#### 4.1.5. Vascular Tissue Engineering and Regeneration

With the ever increasing numbers of patients requiring vascular access due to variety of ailments, including cardiovascular disease, peripheral vascular disease, and ischemia, there is a significant need for the ability not only to understand and regulate vasculature development and differentiation, but also to develop vascular grafts or artificial blood and lymphatic vessels. The field of vascular tissue engineering has undergone considerable progress over the past decade [130]. MC, already widely accepted and used in numerous biomedical applications, have shown to represent a valid alternative scaffold material for vascular tissue engineering, as discussed below.

Wang et al. [131] developed a snakehead fish scale-derived collagen patch by lyophilization and cold-pressing of methylated collagen followed by 1,4-butanediol diglycidyl ether (BDE), a well-studied bifunctional crosslinking epoxy compound. In this study, collagen was first modified by methylation to eliminate its carboxylic acid groups via partial esterification, by forming methyl ester groups, producing a net positively charged methylated collagen, which can improve cell interaction by attracting negatively-charged proteins onto the cell membrane [132,133]. In addition, it was reported that methylated collagen is water-soluble and can potentially be used as a drug carrier [134,135]. Next, the methylated collagen was cross-linked with BDE to improve its physicochemical properties, since the common chemical cross-linkers, particularly glutaraldehyde, can potentially lead to cytotoxicity and poor biocompatibility [136]. In this study, favorable integration of the collagen patches into the surrounding tissues, with good infiltration of cells, blood vessels, and lymphatic vessels was observed in a murine model, demonstrating the potential application of fish scale-derived collagen as a scaffolding material for vascular genesis and for various other biomedical applications. 

Tubular scaffolds of marine source collagen from jellyfish and PLGA were fabricated via freeze-drying and electrospinning for use as vascular grafts [137]. This MC/PLGA composite scaffold not only enhanced the proliferation of primary rabbit smooth muscle cells and endothelial cells and promoted endothelial cell development, but also induced significant directional cell alignment, similar to that of native vessels in vivo, suggesting a promising role of MC/PLGA scaffolds in tissue engineered vascular grafts.

#### 4.1.6. Dental Tissue Engineering and Regeneration

The structure of a tooth is unique; the soft and hard tissues exist together, with the hard tissue covering the soft tissue, the dental pulp [138]. Over the past decade, the field of dental tissue engineering has witnessed tremendous progress toward new concepts of the reparative paradigm, giving rise to the emerging field of regenerative dentistry [139]. 

Type I collagen isolated from tilapia (*Oreochromis niloticus*) scales promoted increased cell viability and attachment, enhanced alkaline phosphatase activity, and upregulated the gene expression of bone sialoprotein in rat odontoblast-like cells (MDPC-23), as well as accelerated matrix mineralization, with effects comparable to those obtained with porcine skin, proposing fish collagen’s applicability in the dental field for use in dentin-pulp regeneration [140]. 

The role of MCP in osteogenic differentiation was first shown by reports that MCP promoted viability of primary human periodontal ligament (hPDL) cells and upregulated the expression of osteogenic markers and osteogenesis-related proteins via the ERK signaling pathways, suggesting that MCP is a promising bioactive ingredient for biomaterials used in alveolar bone regeneration [141]. A biomimetic electrospun tilapia fish collagen/bioactive glass/chitosan nanofiber membrane showed some level of anti-bacterial activity against *Staphylococcus mutans* and enhanced cell adhesion, cell viability, and the expression of osteogenic genes in hPDL cells [142]. This scaffold also promoted bone regeneration in periodontal defect dog models, demonstrating its potential for clinical application as a guided tissue or bone regeneration membrane for inducing periodontal tissue regeneration. 

#### 4.1.7. Corneal Tissue Engineering and Regeneration 

Corneal damage is one of the leading causes of blindness worldwide [143]. Corneal transplantation (keratoplasty) is the definitive treatment when vision cannot be corrected using other treatments. Corneal transplantation is the most commonly performed solid tissue transplant procedure in the world [144]. However, a severe shortage of donor corneas is the greatest challenge in this field. Although different biomaterials, including amniotic membranes, acellular corneal stroma, and natural polymer-based materials have been used as the cornea repair materials and were found to have excellent biocompatibility and support epithelization, few studies have focused on corneal stromal wound healing with regards to scar formation and transparency improvement [145]. Collagen-based biomaterials were successfully used to achieve corneal repair and scar inhibition [146].

A decellularized and decalcified fish scale-derived collagen matrix was proposed as an alternative for human donor corneal tissue [147]. Ocular implantation of the MC-based matrix in a rat model of anterior lamellar keratoplasty showed good biocompatibility, adequate light transmission, reasonable light-scattering values, and the ability to be used in keratoplasty, suggesting the feasibility of MC for use in corneal replacement to combat the shortage of donor corneas. When compared with a denuded human amniotic membrane, MC-based scaffolds synthesized from fish scales via freeze-drying displayed good mechanical and physical strengths, a better swelling ratio, greater microbial resistance, and enhanced viability, growth, proliferation, and migration of limbal epithelial cells from limbal explants, supporting MC as a potential biomaterial candidate for corneal regeneration or transplantation [148].

#### 4.1.8. Other Tissue Engineering and Regeneration Data

Over the last few decades, researchers have attempted to develop a human skin substitute for treating full-thickness skin wounds. There is a similar need to provide an oral mucosa substitute, suitable for reconstructing primary or secondary oral mucosal defects due to limited viable donor-derived oral mucosal soft tissues. An ex vivo-produced oral mucosa equivalent has been successfully constructed and used for reconstruction of oral mucosal defects derived from oncologic resection, vestibuloplasty, and periodontal therapy [149,150,151]. An MC/chitosan composite scaffold was fabricated using isolated tilapia scale collagen via the process of lyophilization and dehydrothermal cross-linking at 130 °C in vacuo for use as a tissue engineered oral mucosa equivalent [152]. Primary oral keratinocytes cultured on this composite scaffold produced a multilayered, polarized, stratified epithelial layer with superficial keratinization, demonstrating its potential application in epithelial tissue engineering and its effectiveness as a new potential therapeutic device in oral mucosa regenerative medicine. 

A bioactive MC/polycaprolactone composite nanofibrous scaffold was fabricated via electrospinning for 3D cell culture of mouse thymic cortical epithelial reticular cells and this scaffold was shown to not only facilitate cell adhesion, spreading, protrusions, and proliferation, but also to stimulate the expression of genes and proteins involved in cell adhesion and T-cell development, suggesting that the scaffold has applicability in epithelial tissue or lymphoid tissue engineering [153].

### 4.2. Drug Delivery 

The use of large sized materials in drug delivery poses major challenges, including in vivo instability, poor bioavailability, solubility, and poor absorption into the body tissues with target-specific delivery and tonic effectiveness, and probable adverse effects. Therefore, using new drug delivery systems for targeting drugs to specific body parts could be an option that might solve these critical issues. Hence, nanotechnology plays a significant role in advanced medicine/drug formulations and their controlled drug release and delivery has been an immense success [154]. Several studies have shown that collagen could be used as a carrier in these drug delivery systems.

To design a scaffold-controlled release system for skin tissue engineering, silver carp skin collagen/chitosan/chondroitin sulfate scaffolds were fabricated by freeze-drying and incorporated with bFGF-loaded PLGA microspheres, which were uniformly distributed into the MC-based loaded scaffolds. The scaffolds exhibited a tunable and tailorable protein release rate, depending on the ratio of collagen to chitosan in the scaffold, and showed good biocompatibility and an ability to promote fibroblast cell proliferation and skin tissue regeneration, thereby demonstrating that this MC-based loaded scaffold can be used in controlled release delivery systems and for wound healing and skin tissue engineering [155]. 

Guo et al. [156] reported simple methods for preparing core-shell MCP chelated calcium/calcium alginate nanoparticles approximately 400 nm in diameter using MCP produced from Synodontidae fish scales and calcium alginate for the encapsulation of calcium. The in vivo experiments indicated that the MCP-based calcium chelated nanoparticles improved calcium absorption and prevented calcium deficiency and also increased femur bone mineral density and calcium content in rats, proposing that MCP-based nanoparticles could be an ideal carrier for use in calcium supplementation. The suitability of the collagen gels and films synthesized using ASC and PSC from the eel fish (*Evenchelys macrura*) skin was tested for carrying drugs, such as ampicillin and tetracycline. It was shown that these collagen gels and films could be used as efficient carriers for anti-bacterial and anti-fungal drugs for use in drug delivery systems [157]. An injectable composite chitosan/marine collagen gel has been developed using atelocollagens from the fresh skin, bone, and scales of chum salmons (*Oncorhynchus keta*) and was injected subcutaneously in rats, where it was observed that the inflammatory cell infiltration and release of tumor necrosis factor-α (TNF-α) were successively controlled. Additionally, the injected gel had been replaced with the fibrous tissue composed of fibroblasts and ECM, indicating this composite gel could be a suitable carrier for tissue filler and drug delivery systems [158].

New bio-based topical formulations for wound exudate absorption were developed using a natural marine sponge skeleton, where the collagenic skeleton of marine sponges acted as biocompatible carrier for the loading of L-cysteine hydrochloride, a sulfur amino acid known for its wound healing properties, suggesting that the natural sponge skeletal scaffold might act as a bioactive-biomimetic carrier facilitating the wound healing processes due to its glycosaminoglycans [43]. A novel gastroresistant delayed-release tablet coating based on the marine sponge *Chondrosia reniformis* collagen was developed and it was found that the tablets coated with sponge collagen resisted exposure to HCl. Disintegration of all tablets occurred in pH 6.8 phosphate buffer solution, demonstrating a suitability of this novel coating in delayed-release tablets, providing good mechanical properties and storage stability [159]. Nanoparticles of the marine sponge *Chondrosia reniformis* collagen were also used as penetration enhancers and were developed by controlled alkaline hydrolysis for the transdermal drug delivery of 17β-estradiol-hemihydrate for hormone replacement therapy [160]. In this study, the hydrogel with estradiol-loaded MC-based nanoparticles enabled prolonged estradiol release compared to a commercial gel and resulted in considerably enhanced estradiol absorption, indicating that marine sponge collagen nanoparticles represent promising carriers for transdermal drug delivery. 

### 4.3. Therapeutic Effects of MC on Diseases Associated with Metabolic Disturbance 

Over the last century, obesity has emerged as a leading global health concern, mainly driven by poor dietary habits such as the consumption of high-calorie or high-fat foods, insufficient physical activity, and an increasingly sedentary lifestyle [161]. Indeed, the global prevalence of obesity nearly doubled between 1980 and 2014, such that approximately 40% of adults worldwide are overweight and 13% are clinically obese. Obesity is known to be the main risk factor not only for a number of non-communicable diseases like type-2 diabetes mellitus (T2DM), cardiovascular disease, hypertension, coronary heart disease, or certain types of cancers but also for diverse psychological problems and various physical disabilities [162]. Despite decades of intense, targeted research, the cures for these life-threatening conditions still remain elusive, and safe and efficacious anti-obesity therapeutics have not yet been developed. There is no advanced candidate with curative potential, not even one with the promise to manage obesity in a fashion comparable to the treatment of diabetes with insulin [163]. Thus, there is a great need for the discovery and development of novel strategies for the management and treatment of serious medical issues associated with metabolic abnormalities. 

It was recently reported that diet-induced obesity and associated disorders can be prevented by natural bioactive warm sea MCP treatment [164]. In this study, MCP facilitated a lower increase in body weight, a weaker increase in fat mass, and a decreased expression of inflammatory cytokines in mice fed with a high fat diet (HFD), paving the route for the potential utilization of MCP in human obesity-associated metabolic disorders. Zhu et al. [165] elucidated the role of MCPs from chum salmon skin in the protection of carotid artery vascular endothelial cells by inhibiting expression of apoptosis biomarkers, attenuating endothelial thinning and inflammatory exudation, and reducing blood glucose levels in a T2DM rat model, providing evidence that MCPs protect against cardiovascular endothelial cell injury and alleviate cardiovascular dysfunction in T2DM. These results suggest that MCPs may be a novel therapeutic tools to protect against early cardiovascular complications associated with T2DM. In line with these results, MCPs were found to improve glucose metabolism and insulin resistance, to decrease the expression of oxidative stress biomarkers and inflammatory cytokines and adipocytokines in livers of T2DM rat models [166]. 

It was reported that MCP significantly reduced levels of fasting blood glucose, fasting blood insulin, total triglycerides, total cholesterol, low-density lipoprotein (LDL), and free-fatty acids, but increased the insulin sensitivity index, as well as levels of high-density lipoprotein (HDL) and adiponectin, indicating that MCPs improved glucose and lipid metabolism and may help control hyperglycemia in T2DM patients by downregulation of chronic inflammation and upregulation of and adiponectin production [167]. MCP also significantly reduced levels of fasting blood glucose, diastolic blood pressure, mean arterial pressure, serum triglycerides, total cholesterol, LDL, free fatty acids, but increased levels of HDL and adiponectin, the insulin sensitivity and insulin secretion indices, indicating that treatment with MCP improves glucose and lipid metabolism profiles, insulin sensitivity, renal function, and hypertension management in patients with T2DM and hypertension, thereby suggesting that MCPs may be used in patients with both T2DM and hypertension [168]. In addition, MCP downregulated levels of free fatty acids, but upregulated levels of adiponectin [169]. Notably, in this study, protection exerted by MCP seemed more profound in individuals who were only diabetic than in diabetics with hypertension, suggesting that MCP could offer protection against diabetes and hypertension by affecting different levels of molecules involved in diabetic and hypertensive pathogenesis. Clinical studies using two commercial MCP products, Nutripeptin^®^ and Hydro MN Peptide^®^, exhibited that they stabilized blood glucose levels, reduced obesity risk, and promoted a prolonged sense of satiety in T2DM patients [105].

Lee et al. [170] investigated the effect of MCP isolated from tuna skin on the adipogenic differentiation of 3T3-L1 preadipocytes and in obese HFD-fed mice. In this study, MCP significantly inhibited lipid accumulation during the differentiation of mouse preadipocytes (3T3-L1), which was accompanied by decreased expression of key regulators of adipocyte differentiation and maintenance. Moreover, MCP suppressed the accumulation of palmitate-induced lipid vacuoles in hepatocytes, reduced adipocyte size, reduced serum levels of total cholesterol, triglyceride, and LDL, but increased serum HDL. These observations suggest that MCP inhibits adipocyte differentiation through a mechanism involving transcriptional repression of the major adipogenic regulators, thereby reducing body weight gain and adipogenesis. The results provide insights into the development of future therapeutic agents for obesity. 

### 4.4. Limitations 

The topic of this systematic review is a fairly new field of research. Only a few clinical trials have been conducted in humans to evaluate the efficacy of MCP products. Although these trials have had some positive results, they have mostly been developed as neutraceuticals. Moreover, few clinical studies have been carried out in humans to assess the therapeutic effects of MC-based scaffolds for clinical translational applications. In addition, the potential language bias in this systematic review should be considered since only English-language literature was studied. Further comprehensive and complete investigation is necessary in order to evaluate the therapeutic potential of MC-based scaffolds and MCP products in numerous medical disciplines, especially including tissue engineering therapies in regenerative medicine and surgery, cell therapies, wound dressings, and drug delivery.

## 5. Conclusions and Future Prospective

Over the past few decades, MC has emerged as a promising biomaterial for biomedical applications due to its natural origin and structural similarity to mammalian collagen, which is abundant in living tissues and is one of the most popularly used biomaterials in tissue engineering. In addition, its biocompatibility, easy extractability, water solubility, safety, and low production costs as well as its biodegradability, anti-microbial activity, and functionality make it an attractive biomaterial for tissue engineering and drug delivery. Moreover, the flexibility of the processing conditions of MC aids in the fabrication of versatile substrates as scaffolds for tissue regeneration or carriers of biological molecules. Due to its characteristics and physicobiochemical properties, it has tremendous potential for use as a scaffold biomaterial in tissue engineering applications including bone, cartilage, skin, vascular, dental, and corneal tissue regeneration, in drug delivery systems, and as a therapeutic, especially for diseases associated with metabolic disturbances, such as obesity and diabetes. Despite all the challenges, MC holds great promise as a biomaterial for developing medical products and therapeutics. 

## Appendix A

**Table A1:** Summary of studies using MC-based scaffolds for bone tissue engineering and regeneration.

Form of MC	Manufacture Technique	Materials	Biological Assessment	Outcomes	Ref
Collagen peptide	Enzymatical hydrolysis	MCP from tilapia scale	Primary rat bone marrow-derived mesenchymal stem cells	1. Promoted cell viability. 2. Upregulated expression of osteogenic markers 3. Upregulated expression of endothelial markers	[84]
Collagen peptide	Enzymatical hydrolysis	MCP from cod bone and skin	Human osteoblastic cells (NOS-1)	1.Promoted cell proliferation 2. Upregulated expression of osteogenic markers 3. Accelerated matrix mineralization	[85]
Collagen peptide	Enzymatical hydrolysis	MCP from Gadiformes and Pleuronectidae	Mouse pre-osteoblastic cells (MC3T3-E1)	1. Upregulated expression of collagen modifying enzymes 2. Greater collagen deposition 3. Accelerated matrix mineralization	[86]
Native collagen	Freeze-drying	Tilapia scale collagen	Primary human mesenchymal stem cells	1. Accelerated early stage of osteoblastic differentiation 2. Upregulated osteoblastic markers	[87]
Collagen peptide	Enzymatical hydrolysis	MCP from chum salmon skin	In vivo rat model	1. Increased size, weight, and mineral density and content of femurs 2. Enhanced stiffness and toughness of femurs	[88]
Collagen peptide	Enzymatical hydrolysis	MCP from Sparidae and Chanos	Human osteoblast-like cells (MG-63)	1. Increased osteoblast proliferation 2. Inhibited osteoclast proliferation	[89]
Scaffold	Freeze-drying/EDC cross-linked	Fish scale collagen from Rohu and Catla	Mouse fibroblasts (NIH3T3)/human osteoblast-like cells (MG-63)/in vivo mouse model	1. Promoted cell proliferation 2. Elicited minimal inflammatory response	[91]
Scaffold	Freeze-drying/EDC/NHS or HMDI cross-linked	Shark skin collagen/shark teeth apatite	Human osteosarcoma cells (Saos-2)	Increased cell viability	[92]
Scaffold	Freeze-drying	Marine sponge collagen/chitosan/hydroxyapatite	Human osteoblast-like cells (MG-63)	Promoted cell proliferation	[93]
Scaffold	Freeze-drying/glutaraldehyde cross-linked	Type-II collagen from shark cartilage/chitosan/hydroxyappatite	Human fetal osteoblasts/human acute T-lymphocyte leukemia cells (6T-CEM)	1. Increased cell viability 2. Enhanced alkaline phosphatase activity	[94]
Scaffold	Freeze-drying/dehydrothermal treatment cross-linked	MC/glycosaminoglycan/Aquamin	Mouse pre-osteoblastic cells (MC3T3-E1)	Improved mineralization	[95]
Scaffold	Vacuum drying/PMMA aggregated	Marine sponge collagen/hydroxyapatite/poly (methyl methacrylate)	Mouse pre-osteoblastic cells (MC3T3-E1)/mouse fibroblasts (L929)	Promoted cell viability	[96]
Scaffold	Glutaraldehyde/genipin cross-linked	Sturgeon fish collagen/poly (N,N’-dimethylacrylamide	In vivo rabbit bone defect model	1. Good biomechanical performance 2. Strong bonding ability with bone	[97]
Scaffold	Freeze-drying/EDC cross-linked	Mineralized salmon collagen/alginate/fibrillized jellyfish collagen	Primary bone marrow-derived mesenchymal stem cells	Induced osteogenic and chondrogenic differentiation	[98]
Scaffold	Electrospinning	Fish collagen/PLGA/hydroxyapatite	Primary bone marrow-derived mesenchymal stem cells/human gingiva fibroblasts	1. Enhanced mechanical strength and the degradation rate 2. Improved cytocompatibility	[99]

**Table A2:** Summary of studies using MC-based scaffolds for cartilage tissue engineering and regeneration.

Form of MC	Manufacture Technique	Materials	Biological Assessment	Outcomes	Ref
Collagen peptide	Enzymatical hydrolysis	MCP from skin of deep water ocean fish (cod, haddock and pollock).	Primary horse adipose-derived stromal cells	1. Increased glycosaminoglycan expression 2. Induced chondrogenic differentiation	[100]
Collagen peptide	Enzymatical hydrolysis	MCP from skins of Gadiformes	In vivo rabbit osteoarthritis model	Chondroprotective effects	[101]
Native collagen	Acid soluble collagen isolation method	Tilapia fish scale collagen	Human mesenchymal stem cells	1. Increased glycosaminoglycan expression 2. Elevated expression of chondrogenic markers 3. Enhanced chondrogenic differentiation	[102]
Scaffold	Freeze-drying/EDC cross-linked	Jellyfish collagen	Primary human and rat nasal septum chondrocytes/in vivo rat septal cartilage defect model	1. Promoted adhesion and cartilaginous matrix proteins production 2. Reduced nasal septum perforations	[103]
Scaffold	Freeze-drying/EDC cross-linked	Fibrillized jellyfish collagen/alginate	Primary human mesenchymal stem cells	Induced chondrogenic differentiation	[104]
Collagen peptide	Enzymatical hydrolysis	Pharmaceutical grade collagen hydrolysate	Clinical studies in ortheoarthritic patients	1. Cartilage matrix synthesis 2. Reduced pain	[105]

**Table A3:** Summary of studies using MC-based scaffolds for skin tissue engineering and regeneration, and wound healing.

Form of MC	Manufacture Technique	Materials	Biological Assessment	Outcomes	Ref
Scaffold	Solvent casting/glutaraldehyde cross-linked	Aminated poly(3-hydroxybutyrate-co-4-hydroxybutyrate)/tilapia fish skin collagen peptides	Mouse fibroblasts (L929)/in vivo rat wound model	1. Enhanced cell attachment and proliferation 2. Accelerated wound contractions	[110]
Scaffold	Freeze-drying/glutaraldehyde cross-linked	Mrigal fish scale collagen	Primary human fibroblasts and keratinocytes/in vivo rat wound model	1. Enhanced cell growth, attachment, and proliferation 2. Increased wound healing rate, re-epithelialization, and dermal reconstitution	[111]
Scaffold	Freeze-drying/dehydrothermal treatment at 105 °C	Tilapia fish scale collagen/shrimp shell chitosan/glycerin	Primary human keratinocytes and fibroblasts	1. Cytocompatible 2. Facilitated cell proliferation, adhesion, and infiltration	[112]
Scaffold	EDC cross-linked	PSC isolated from catfish skin	Mouse fibroblasts (NIH/3T3)	1. Aligned collagen fibrils 2. Facilitated cell proliferation and migration	[113]
Scaffold	Freeze-drying/EDC cross-linked	Flatfish skin collagen/alginate/chitooligosaccharides	Primary human dermal cells	1. Induced cell adhesion and proliferation 2. Promoted well spread cell morphology	[114]
Scaffold	Freeze-drying	Fish scale collagen	Hamster kidney fibroblasts (BHK21)	Increased cell viability	[115]
Scaffold	Freeze-drying	ASC and PSC from tilapia skin	In vivo rat wound model	1. Increased wound contraction 2. Reduced inflammatory reaction 3. Enhanced collagen synthesis and dermal reconstitution 4. Accelerated epithelization and wound healing	[116]
Scaffold	Freeze-drying/EDC cross-linked	Weever skin collagen/chitosan	Mouse embryonic fibroblasts (MEF)/in vivo rabbit wound model	1. Biocompatible 2. Increased cell proliferation 3. Reduced inflammation 4. Enhanced tissue regeneration and healing	[117]
Collagen peptide	Enzymatical hydrolysis	MCP from Nile tilapia skin	Human keratinocyte (HaCaT)/in vivo rabbit scald wound model	1. Increased cell proliferation 2. Enhanced wound healing	[118]
Collagen peptide	Enzymatical hydrolysis	MCP from chum salmon skin	In vivo rat wound model	Accelerated wound healing	[119]
Collagen peptide	Enzymatical hydrolysis	MCP from chum salmon skin	In vivo rat wound model	1. Faster wound closure and improved tissue regeneration 2. Improved angiogenesis 3. Increased deposition of organized collagen fibers	[120]

**Table A4:** Summary of studies using MC-based scaffolds for wound dressing and skin repair.

Form of MC	Manufacture Technique	Materials	Biological Assessment	Outcomes	Ref
Scaffold	Electrospinning	Tilapia skin collagen/bioactive glass	Human keratinocytes (HaCaT)/primary human dermal fibroblasts/primary human umbilical vein endothelial cells	1. Antibacterial activity against *Staphylococcus aureus* 2. Promoted cell adhesion, proliferation and migration 3. Induced secretion of type I collagen and vascular endothelial growth factor 4. Accelerated skin wound healing in in rat wound model	[122]
Native collagen	Casting-solvent evaporation technique	Marine sponge collagen	Swelling behavior/fluid uptake performance test	1. Suitable swelling behavior and great fluid uptake ability 2. Effective carrier for L-cysteine hydrochloride	43]
Scaffold	Freeze-drying/ceftazidime cross-linked	Aminated carboxymethyl guar gum/fish scale collagen	Mouse fibroblasts (NIH3T3)	1. Excellent biocompatibility 2. Antimicrobial activity against *Staphylococcus aureus* and *Pseudomonas aeruginosa*	[123]
Scaffold	Freeze-drying/glutaraldehyde cross-linked	Fish scale collagen/bean extracts	Mouse fibroblasts (NIH3T3)/human keratinocytes (HaCaT)	1. Excellent biocompatibility with fibroblasts and keratinocytes 2. Good antimicrobial activity and drug release pattern	[124]
Scaffold	Electrospinning	MCP/chito-oligosaccharides	Human skin fibroblasts	1. Good antibacterial activities against *Staphylococcus aureus* and *Escherichia coli* 2. Supported fibroblast proliferation	[125]
Collagen peptide	Enzymatical hydrolysis	Commercially available fish type I collagen hydrolysate from Amino collagen (Meiji Seika, Tokyo, Japan)	6 weeks clinical studies in 25 Japanese women volunteers (35.1 ± 5.4 years old)	Improved skin hydration	[126,127]
Collagen peptide	Enzymatical hydrolysis	MCP stabilized orthosilicic acid	Randomized patient groups	1. No side effects, hypersensitivity, or systemic symptoms 2. Skin rejuvenation	[128]
Collagen peptide	Enzymatical hydrolysis	Marine sponge collagen	Mouse fibroblasts (L929)/human keratinocytes (HaCaT)	1. Increased cell proliferation 2. Photo-protective	[129]

**Table A5:** Summary of studies using MC-based scaffolds for vascular tissue engineering and regeneration.

Form of MC	Manufacture Technique	Materials	Biological Assessment	Outcomes	Ref
Scaffold	Freeze-drying/cold-pressing/1,4-butanediol diglycidyl ether cross-linked	Snakehead fish scale collagen	Mouse lymphatic endothelial cells	1. Improved cell attachment, proliferation and infiltration 2. Favorable growth of blood and lymphatic vessels	[131]
Scaffold	Electrospinning	Acid-soluble jellyfish collagen/PLGA	Primary rabbit aortic endothelial cells and smooth muscle cells	1. Enhanced cell proliferation 2. Directional cell alignment 3. Upregulated expressions smooth muscle and endothelial cell activity-related molecules 4. Enhanced endothelial cell development, and retention of the differentiated cell phenotype	[137]

**Table A6:** Summary of studies using MC-based scaffolds for dental tissue engineering and regeneration.

Form of MC	Manufacture Technique	Materials	Biological Assessment	Outcomes	Ref
Collagen peptide	Enzymatical hydrolysis	Tilapia scale type I collagen	Rat odontoblast-like cells (MDPC-23)	1. Increased cell viability and cell attachment 2. Enhanced osteogenic gene expression 3. Accelerated matrix mineralization	[140]
Collagen peptide	Enzymatical hydrolysis	MCP from tilapia scales	Primary human periodontal ligament cells	1. Promoted cell viability 2. Upregulated expression of osteogenic markers and osteogenic-related proteins	[141]
Scaffold	Elecrospinning	Tilapia fish collagen/bioactive glass/chitosan	Primary human periodontal ligament cells/in vivo dog furcation defect model	1. Antibacterial activity on *Streptococcus mutans* 2. Enhanced viability and osteogenic differentiation 3. Promoted bone regeneration	[142]

**Table A7:** Summary of studies using MC-based scaffolds for corneal tissue engineering and regeneration.

Form of MC	Manufacture Technique	Materials	Biological Assessment	Outcomes	Ref
Scaffold	Decellularization/decalcification	Tilapia fish scale–derived collagen matrix (FSCM)	In vivo rat ocular implantation model	1. Biocompatible 2. Adequate light transmission 3. Reasonable light-scattering values	[147]
Native collagen	Drying at 25 °C	Seabass scale collagen	Primary human limbal epithelial cells	1. Good swelling ratio and microbial resistance 2. Enhanced cell viability, growth, proliferation, and migration	[148]

**Table A8:** Summary of studies using MC-based scaffolds for other types of tissue engineering and regeneration.

Form of MC	Manufacture Technique	Materials	Biological Assessment	Outcomes	Ref
Scaffold	Freeze-drying/dehydrothermal cross-linked	Tilapia fish scale collagen/chitosan	Primary oral keratinocytes	Produced multilayered, polarized, stratified epithelial layer with superficial keratinization	[152]
Scaffold	Electrospinning	MCP from tilapia fish scale/PCL	Thymic epithelial cells	1. Facilitated cell adhesion, spreading, protrusions, and proliferation 2. Stimulated expression of thymopoietic genes and proteins	[153]

**Table A9:** Summary of studies using MC-based scaffolds for drug delivery.

Form of MC	Manufacture Technique	Drug	Biological Assessment	Route	Ref
MC based Scaffolds/PLGA microspheres	Silver carp skin collagen/chitosan/chondroitin sulfate/PLGA	Basic fibroblast growth factor	In vivo rat full-thickness skin wound model	Implanted subcutaneously	[155]
Nanoparticle	Synodontidae fish scale collagen/calcium alginate	Calcium	Calcium content and bone mineral density	Intragastric administration	[156]
Gels/films	Eel skin collagen	Antimicrobial drugs (ampicillin and tetracycline)	Anti-bacterial activity (*Klebsiella pneumoniae, Staphylococcus aureus, Vibrio cholera,* and *Pseudomonas aeruginosa*)/anti-fungal activity (*Epidermophyton floccosum, Trichophyton mentagrophytes,* and *Candida albicans*)	In vitro test	[157]
Injectable gel	Collagen from chum salmon skin, bone and scales/chitosan	-	In vivo rat model	Implanted subcutaneously	[158]
Powder/polymeric film	Marine sponge collagen	L-cysteine hydrochloride	In vitro permeation study	Topical application	[43]
Coating	Marine sponge collagen	-	Disintegration test	In vitro test	[159]
Nanoparticle	Marine sponge collagen	17β-estradiol-hemihydrate	Transdermal absorption in human study	Topical application	[160]

**Table A10:** Summary of studies on the therapeutic effects of MC.

Form of MC	Source of MCP	Biological Assessment	Administration Route	Outcomes	Ref
Collagen peptide	Warm sea fish skin	In vivo HFD-fed mouse model	Oral	1. Suppressed gain of weight and fat mass 2. Reduced levels of pro-inflammatory cytokines	[164]
Collagen peptide	Chum salmon skin	In vivo T2DM rat model	Oral	1. Inhibited expression of apoptosis biomarkers 2. Attenuated endothelial thinning and inflammatory exudation 3. Reduced blood glucose levels	[165]
Collagen peptide	Chum salmon skin	In vivo T2DM rat model	Oral	1. Improved glucose metabolism and insulin resistance 2. Decreased expression of oxidative stress biomarkers, inflammatory cytokines, and adipocytokines	[166]
Collagen peptide	Chum salmon skin	Clinical study in patients with T2DM	Oral	1. Reduced levels of fasting blood glucose, fasting blood insulin, total triglycerides, total cholesterol, LDL, and free-fatty acids 2. Increased levels of insulin sensitivity index, HDL, and adiponectin	[167]
Collagen peptide	Chum salmon skin	Clinical study in patients with T2DM and primary hypertension	Oral	1. Reduced levels of fasting blood glucose, diastolic blood pressure, mean arterial pressure, serum triglycerides, total cholesterol, LDL, and free-fatty acids 2. Increased levels HDL, adiponectin, insulin sensitivity index, and insulin secretion index	[168]
Collagen peptide	Chum salmon skin	Clinical study in patients with T2DM and primary hypertension	Oral	1. Decreased levels of free fatty acid 2. Increased levels of adiponectin	[169]
Collagen peptide	Fish protein hydrolysate	Clinical study of two commercial MCP products (Nutripeptin^®^ and Hydro MN Peptide^®^) in patients with T2DM	Oral	1. Stabilized blood glucose levels 2. Reduced obesity risk 3. Promoted a prolonged sense of satiety	[105]
Collagen peptide	Tuna skin	Mouse preadipocytes (3T3-L1)/in vivo HFD-fed mouse obesity model	Oral	1. Inhibited lipid accumulation 2. Decreased expressions of key regulators in adipocyte differentiation and maintenance 3. Suppressed accumulation of palmitate-induced lipid vacuoles in hepatocytes 4. Reduced adipocyte size. 5. Reduced serum levels of total cholesterol, triglyceride, and LDL 6. Increased serum level of HDL	[170]
